# Vaccine-Derived Poliovirus Infection among Patients with Primary Immunodeficiency and Effect of Patient Screening on Disease Outcomes, Iran

**DOI:** 10.3201/eid2511.190540

**Published:** 2019-11

**Authors:** Mohammadreza Shaghaghi, Shohreh Shahmahmoodi, Ali Nili, Hassan Abolhassani, Seyedeh Panid Madani, Ahmad Nejati, Maryam Yousefi, Yaghoob M. Kandelousi, Mona Irannejad, Shiva Shaghaghi, Seyed Mohsen Zahraei, Sussan Mahmoudi, Mohammad Mehdi Gouya, Reza Yazdani, Gholamreza Azizi, Nima Parvaneh, Asghar Aghamohammadi

**Affiliations:** Johns Hopkins Hospital, Baltimore, Maryland, USA (M. Shaghaghi);; Tehran University of Medical Sciences, Tehran, Iran (M. Shaghaghi, S. Shahmahmoodi, A. Nili, H. Abolhassani, S.P. Madani, A. Nejati, M. Yousefi, Y.M. Kandelousi, M. Irannejad, S. Shaghaghi, R. Yazdani, N. Parvaneh, A. Aghamohammadi);; Research Center for Immunodeficiencies, Tehran (M. Shaghaghi, A. Nili, H. Abolhassani, S.P. Madani, M. Irannejad, S. Shaghaghi, R. Yazdani, N. Parvaneh, A. Aghamohammadi);; Karolinska University Hospital Huddinge, Stockholm, Sweden (H. Abolhassani);; Ministry of Health and Medical Education, Tehran (S.M. Zahraei, S. Mahmoudi, M.M. Gouya);; Alborz University of Medical Sciences, Karaj, Iran (G. Azizi);; Non-Communicable Diseases Research Center, Karaj (G. Azizi)

**Keywords:** poliomyelitis, vaccine, paralysis, primary immunodeficiency, stem cell transplantation, vaccine-derived poliovirus, vaccine-preventable diseases, viruses, Iran, patient screening, poliovirus

## Abstract

Patients with immunodeficiency-associated vaccine-derived poliovirus (iVDPV) are potential poliovirus reservoirs in the posteradication era that might reintroduce polioviruses into the community. We update the iVDPV registry in Iran by reporting 9 new patients. In addition to national acute flaccid paralysis surveillance, cases were identified by screening nonparalyzed primary immunodeficiency (PID) patients. Overall, 23 iVDPV patients have been identified since 1995. Seven patients (30%) never had paralysis. Poliovirus screening accelerated the iVDPV detection rate in Iran after 2014.The iVDPV infection rate among nonparalyzed patients with adaptive PID was 3.1% (7/224), several folds higher than previous estimates. Severe combined immunodeficiency patients had the highest risk for asymptomatic infection (28.6%) compared with other PIDs. iVDPV2 emergence has decreased after the switch from trivalent to bivalent oral poliovirus vaccine in 2016. However, emergence of iVDPV1 and iVDPV3 continued. Poliovirus screening in PID patients is an essential step in the endgame of polio eradication.

During the 3 decades since the establishment of the Global Polio Eradication Initiative (GPEI), the use of oral poliovirus vaccine (OPV) and inactivated poliovirus vaccine (IPV) has led to a >99.99% decrease in the incidence of wild-type poliomyelitis (≈350,000 cases in 1988 to 32 cases in 2018) and eradication of wild poliovirus type 2 (WPV2) (*1*–*4*). No report on wild poliovirus type 3 has occurred since 2012. WPV1 is still circulating in some areas of Pakistan, Afghanistan, and Nigeria (*5*,*6*). OPV has been the backbone of eradication strategy as an inexpensive and easily accessible tool (*7*). However, its widespread use has been associated with some adverse events, including vaccine-associated paralytic poliomyelitis and the emergence of vaccine-derived polioviruses (VDPVs) (*8*). To date, most VDPV case-patients excreted serotype 2 viruses in stool (*9*,*10*). Accordingly, the World Health Organization (WHO) changed the worldwide immunization schedules to end the administration of trivalent OPV (tOPV), which includes serotypes 1, 2, and 3, and introduce bivalent OPV (bOPV), which includes serotypes 1 and 3, alongside >1 dose of IPV (all 3 serotypes) (*11*,*12*).

The genome of OPV strains is susceptible to spontaneous mutations because of its unstable structure, and the emergence of VDPVs threatens the whole eradication program (*13*). By definition, VDPV serotypes 1 and 3 have >1% divergence in the viral protein (VP) 1 coding region of their original OPV strain. VDPV2 is defined when the OPV strain serotype 2 attains >0.6% VP1 nucleotide divergence (*10*,*14*). Patients with primary immunodeficiencies (PIDs) are susceptible to not clearing the vaccine strains after receipt of OPV, which provides an environment for prolonged virus replication and genomic changes. These patients have an ≈3,000-fold increased risk for onset of prolonged immunodeficiency-associated VDPV (iVDPV) infection and vaccine-associated paralysis (*2*).

In previous studies, we reported the largest series of patients who had paralysis because of iVDPV infection (*1*). Given the implementation of poliovirus screening programs on PID patients, the number of nonparalyzed iVDPV excretors has increased substantially in recent years (*9*). In this study, we update our registry of patients in Iran with iVDPV infection. In addition to describing important clinical and virologic properties of newly identified patients, our findings underscore the importance of poliovirus screening programs in increasing the iVDPV detection rate.

## Study Design

We collected data on patients with iVDPV shedding up to the end of 2018 by consulting a national registry of patients with acute flaccid paralysis (AFP) and by screening the stool of nonparalyzed PID patients for poliovirus infection. All AFP case-patients in Iran are routinely examined for shedding of polioviruses. Stool specimens were collected within 14 days after paralysis onset.

In our screening program, virologic data of patients were collected prospectively during 2014–2018. Patients with an established PID diagnosis recorded in our national PID registry and without any paralytic symptom were considered eligible (*15*,*16*). Inclusion criteria were not restricted to patients’ age or any specific PID type. We also included patients with innate immunodeficiencies because these patients might be exposed to iVDPV-excreting patients during hospital stays. Patients consented to and were recruited for 1-time poliovirus screening at admission to the healthcare facilities related to our registry network as well as for routine follow-up visits, intravenous immunoglobulin (IVIG) infusion, or infection control. Our method for stool sampling has been described previously (*15*). Patients were screened only once unless they had positive results in their first encounter, in which case they continued monthly stool testing until the infection cleared. In each encounter, 2 stool specimens were collected and tested over a 4-day period, depending on the proximity of the patient to the study site.

All stool specimens were processed at Iran’s National Polio Laboratory in Tehran by using cell culture and real-time reverse transcription PCR, in accordance with WHO protocol (*17*). Poliovirus isolates were sent to the US Centers for Disease Control and Prevention (Atlanta, Georgia, USA) for sequencing of the VP1 genomic region.

For our analysis, we defined VP1 divergence rate as the maximum value (percentage) of VP1 divergence from the original Sabin strain, divided by the total duration (years) of virus replication. As an index for a timely detection of infection, we calculated iVDPV detection speed as the duration of observed iVDPV excretion divided by the total time of iVDPV replication (*9*). To calculate these parameters, we considered the time interval between the first OPV administration and the last iVDPV isolation as the approximate duration of virus replication (*9*).

We extracted clinical and immunologic data from our national PID registry database (*16*,*18*). We categorized patients into a combined immunodeficiency (CID) group (including severe CID [SCID], less severe CID [e.g., major histocompatibility complex class II deficiency], and CID with syndromic features); a predominantly antibody deficiency (PAD) group (including agammaglobulinemia [autosomal and X-linked] and hypogammaglobulinemia); or an innate immunodeficiency group (e.g., neutropenia, chronic granulomatous disease, and complement deficiency), on the basis of the diagnostic criteria of the European Society for Immunodeficiencies (https://esid.org/Working-Parties/Registry-Working-Party/Diagnosis-criteria). We performed genetic evaluation on genomic DNA extracted from whole blood, as described previously (*19*,*20*).

For patients with classical clinical profiles suggestive of a specific CID and agammaglobulinemia, we performed Sanger sequencing on the most likely genes. For patients with failed Sanger sequencing or with clinical characteristics of hypogammaglobulinemia resembling several genetic defects, we performed targeted next-generation sequencing and whole-exome sequencing by using a pipeline described previously (*19*). We reevaluated the pathogenicity of all disease-attributable gene variants by using the updated guideline for interpretation of molecular sequencing by the American College of Medical Genetics and Genomics as described previously (*19*). We performed monthly collection and analysis of stool specimens until the clearance of infection or death. By definition, clearance of iVDPV was achieved when stool was poliovirus-negative for 2 consecutive months (*1*). 

We used Stata 14 software (https://www.stata.com) for descriptive and analytical statistics, the Shapiro-Wilk test to assess the normality of distributions, independent *t*-tests for parametric and Mann-Whitney U tests for nonparametric assessment of associations, and the Pearson χ^2^ test for categorical parameters. We considered a p value <0.05 statistically significant.

## Results

### Symptomatic iVDPV-Excreting Patients

We identified 16 patients who had AFP and concurrent iVDPV shedding during 1995–2016 (Appendix Table). Patients 1–14 have been thoroughly described in previous reports (*1*,*21*). Two new cases with paralysis were identified after 2014.

The first paralysis patient (patient 17) was a girl who had right leg paralysis at 6 months of age. PID was suspected on the basis of a history of recurrent respiratory tract infections and axillary bacillus Calmette-Guérin lymphadenitis. She was diagnosed with T-B-NK+ SCID. iVDPV serotype 2 with 9 VP1 nucleotide substitutions was isolated from stool. The last stool specimen collected 4 months after paralysis was positive for iVDPV2 and had 14 nucleotide substitutions. Her parents and monozygotic twin sister, who also had SCID and had received 3 OPV doses, had poliovirus-negative stool. The patient died at age 1 year.

The second paralysis patient (patient 21) was a 14-month-old boy who had seizure and muscle weakness in the neck, trunk, lower limbs, and right upper limb. Low serum immunoglobulins and B-lymphopenia evinced the diagnosis of agammaglobulinemia. iVDPV serotype 2 was isolated from stool. Stool collected 3 months later was poliovirus-negative. The patient is now in good health and is receiving regular IVIG, having only residual paralysis in his right leg.

### Asymptomatic iVDPV-Excreting Patients

**Figure 1:**
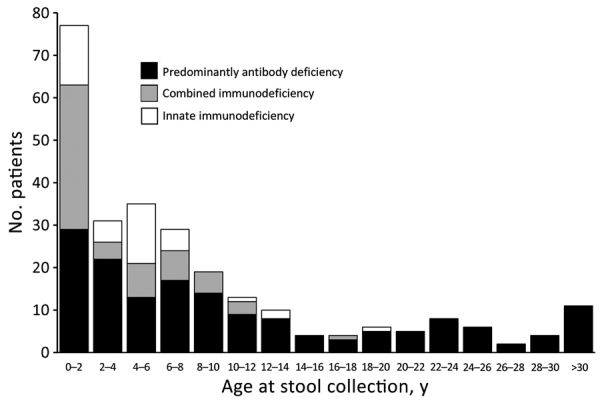
Patient ages at the time of first stool screening in study of vaccine-derived poliovirus infection among patients with primary immunodeficiency, by category of primary immunodeficiencies, Iran, 1995–2018.

Since the beginning of our poliovirus screening program among PID patients, a total of 266 nonparalyzed patients (175 boys and 91 girls) have been evaluated. Median age (interquartile range [IQR]) was 5.0 (1.3–11.0) years in all patients, 7.5 (3.0–18.5) years in patients with PAD, 1.4 (0.9–6.0) years in patients with CID, and 4.5 (1.0–5.2) years inpatients with innate immunodeficiencies (Figure 1). A total of 602 stool specimens were collected from these patients; 58 specimens were collected from iVDPV-excreting patients during their shedding periods. The most frequent adaptive PID was CVID (76 patients [28.6%]), followed by agammaglobulinemia (51 patients [19.2%]) and SCID (21 patients [7.9%]). Forty-two (15.79%) patients had defective innate immunity (Table).

**Table:** Patients Age, PID type, and stool screening results in nonparalyzed PID patients in study of vaccine-derived poliovirus infection among patients with PID, Iran, 1995–2018*

Immunodeficiency	No. patients (%), N = 266	Age at stool collection, y, median (IQR)	Stool screening result
Predominantly antibody deficiencies			
AGG	51 (19.17)	5.0 (2.5–10.7)	1 iVDPV, 3 SL, 1 NPEV
CVID	76 (28.57)	12.5 (7.0–20.7)	2 SL, 3 NPEV
HIGM	17 (6.39)	6.8 (3.2–9.5)	Negative
HGG	18 (6.77)	3.0 (1.2–7.0)	1 SL
Combined immunodeficiencies			
SCID	21 (7.89)	0.9 (0.7–1.2)	5 iVDPVs, 1 SL
Less severe CIDs	21 (7.89)	1.5 (1.0–4.8)	1 iVDPV, 3 SL
CID with syndromic features	20 (7.52)	6.5 (5.0–8.7)	Negative
Innate immunity defects	42 (15.79)	4.5 (1.0–5.2)	2 SL

*AGG, agammaglobulinemia; CID, combined immunodeficiency; CVID, common variable immunodeficiency; HGG; unspecified hypogammaglobulinemia; HIGM, hyper-immunoglobulin M syndrome; IQR, interquartile range; iVDPV, immunodeficiency-associated vaccine-derived poliovirus; MHC2, major histocompatibility class 2 deficiency; NPEV, nonpolio enterovirus; PID, primary immunodeficiency; SL, Sabin-like poliovirus; SCID, severe combined immunodeficiency.

Four (1.5%) patients were excreting nonpolio enteroviruses, and 12 (4.5%) were excreting Sabin-like (SL) polioviruses only. These patients were retested in the second encounter after 1 month; all had negative results. Seven patients (2.6%) were found to shed iVDPVs and did not have symptoms of paralysis. Of note, 6 of these patients were detected among SCID case-patients (6/21 [28.6%]) and the other among agammaglobulinemia case-patients (1/51 [2%]). In a previous study, we briefly reported 3 of these patients (patients 16, 18, and 19), who were identified among 102 patients screened during January 2014–November 2015 (*15*). We continued our poliovirus screening program to include 164 additional cases. Four patients (patients 15, 20, 22, and 23) were found to have nonparalytic iVDPV shedding after November 2015.

Patient 15 was a boy with T-B-NK+ SCID identified at 8 months of age. He shed iVDPV1 for several consecutive months and ultimately cleared the infection after a successful hematopoietic stem cell transplantation (HSCT) at age 1 year. Detailed characteristics of this patient have been discussed in a separate study (*22*).

Patient 16 was a girl with several hospitalizations for fever, bacillus Calmette-Guérin adenitis, and recurrent urinary infections. At 8 months old, SCID was diagnosed and confirmed by finding a homozygous nonframeshift trinucleotide deletion in a class II major histocompatibility complex transactivator gene. iVDPV2 with 12 nucleotide substitutions were isolated from her stool when she was 10 months old. During >3 years of continued shedding, her poliovirus evolved to have 37 VP1 nucleotide substitutions. Despite regular IVIG administration, she still had the infection at the latest follow-up testing. She never had paralysis and is now is a candidate for HSCT.

Patient 18 was an infant boy with oral candidiasis, cutaneous manifestations, and failure to thrive. A presumptive diagnosis of SCID was confirmed by detecting a homozygous mutated adenosine deaminase gene. Stool was positive for an iVDPV2 with 6 VP1 nucleotide substitutions. The patient died at 3.5 months of age from a severe respiratory infection.

Patient 19 was a girl admitted to a peripheral hospital for chronic diarrhea, oral aphthous ulcers, and failure to thrive at 5 months of age. However, her PID was not detected, and she received her fourth OPV dose at 6 months of age. After further workup at 11 months of age, we established a final diagnosis of SCID by detecting a homozygous missense mutation in a recombination activating gene 1. iVDPV2 was isolated from stool. Despite appropriate medical therapy, she died less than 1 month later.

Patient 20 was a boy with agammaglobulinemia. A stool specimen collected at 13 months of age was positive for iVDPV2 with 18 nucleotide substitutions and for SL type 3 virus (SL3). iVDPV2 shedding continued for 3 months and had 20 VP1 nucleotide substitutions. Specimens collected at 18 months of age only showed iVDPV3 with 22 nucleotide substitutions; no poliovirus type 2 was detected. iVDPV3 evolved to have 24 nucleotide substitutions until the patient was 21 months of age. Stool specimens tested 6 months later were negative for any iVDPV serotype.

Patient 22 was a boy who had received 4 doses of bOPV (serotypes 1 and 3) and IPV. At 7 months of age, CID was diagnosed and later confirmed by detecting a homozygous single nucleotide deletion in the regulatory factor X associated ankyrin-containing protein gene. His initial stool specimen was positive for SL3 and had 9 VP1 nucleotide substitutions. Serial stool testing showed a continuous poliovirus replication for 4 months and a gain of 12 VP1 mutations, which exceeded the cutoff level for our definition for iVDPV3. Stool collected 5 months later was poliovirus-negative.

Patient 23 was a boy, the fourth child born to consanguineous parents in rural areas. There was a history of 2 siblings’ death from Kostmann syndrome and autosomal-recessive polycystic kidney disease. Immunodeficiency was not detected at birth, and he had received 2 doses of bOPV until 4 months of age, when he had severe pneumonia and SCID. Diagnosis was confirmed by finding a mutated recombination activating gene 2. At 6 months of age, iVDPV1 with 10 VP1 mutations were isolated from his stool. He never had paralysis but died from pneumonia within a few weeks.

### Iran’s iVDPV Registry

Since the establishment of Iran’s iVDPV registry, a total of 23 iVDPV-excreting patients have been identified; 7 (30%) never had paralysis. Nineteen (82.6%) of the 23 patients were boys. PID type was confirmed by molecular studies in 10 patients. Thirteen (56%) patients had CID, and 9 (39%) had PAD. SCID was the most frequent PID type (11 cases [48%]), followed by agammaglobulinemia (8 cases [35%]). One patient had an undetermined PID type. One patient had unspecified hypogammaglobulinemia. The mean (+ SD) age at the time of first iVDPV isolation was 10.82 (+ 5.18) months for all patients. This age was older in patients who had paralysis (11.8 + 5.6 months), compared with nonparalyzed patients (8.6 + 3.1 months). However, the observed difference was not statistically significant (p = 0.17).

Eight patients (89%) with PAD and 7 patients (54%) with CID had paralysis (p = 0.083). Thirteen patients died. Eleven patients (47.8%) cleared the infection from stool during the follow-up period. Of note, 8 (89%) PAD patients cleared the infection, compared with only 3 (23%) CID patients (p = 0.002).

Twenty-six iVDPVs were isolated from 23 patients. Three patients simultaneously shed 2 different serotypes (patients 5, 9, and 20). iVDPV2 was the most frequent serotype (69%), followed by serotype 1 (19%) and serotype 3 (12%). The median (IQR) of maximum VP1 divergence was 1.7% (1.3%–2.3%) and of viral replication time was 12.4 (10.7–19.2) months. The median viral evolution rate was 1.66% (1.14–2.2%) per year. We observed no difference in evolution rate between different groups of patients on the basis of PID category and paralysis occurrence (p>0.1). The mean of iVDPV detection speed was higher in nonparalyzed patients than in those with paralysis (0.24 vs. 0.17). However, this difference was not statistically significant (p = 0.433).

## Discussion

In this study, we describe a comprehensive registry of 23 iVDPV-excreting patients identified in Iran since 1995, as an update to our previous report (*1*). Although most patients initially had paralysis, more than one quarter were asymptomatic and were only identified by poliovirus screening. The rate of asymptomatic iVDPV infection in our national study (2.6% overall; 3.1% in adaptive immunity defects) was several folds higher than this rate in a previous multinational report (0.8%) (*15*), despite a comparable proportion of SCID in both studies. Other studies also reported detection rates several times lower than what we observed in nonparalyzed PID patients (*23*–*25*). These differences can be caused by different proportions of PID types among these studies or the predominance of certain genetic defects or other predisposing factors in different countries.

Paralysis was observed more frequently in patients with PAD than with CID, supporting new evidence that hypothesizes the role of cell-mediated reactions in the progression to paralysis (*9*). PAD patients were also more likely to clear the infection. Immune mechanisms contributing to these observations have been discussed in recent studies; cytotoxic interactions can damage poliovirus-infected motor neurons in the anterior horn of the spinal cord (leading to paralysis) and enterocytes in the gastrointestinal tract (leading to infection clearance) (*4*,*9*,*22*).

The predominance of iVDPV2 is consistent with previous studies (*9*,*10*). Although the global switch to bOPV can reduce the emergence of VDPV2 in OPV-using countries (*26*), bOPV still carries the risk for VDPV emergence from 2 other serotypes (*22*). Gradual evolution of iVDPV3 from SL3 in patient 22 is an illustration of this risk and shows the importance of serial stool testing in immunodeficient SL excreters. According to WHO, iVDPV3 was the most frequent serotype (7/14 iVDPVs) detected worldwide during January 2017–June 2018 (*10*). Asymptomatic shedding of any iVDPV serotype endangers the whole polio eradication program.

A thorough nucleotide analysis revealed that the VP1 evolution rate occasionally exceeded the estimated rate of 1%–2% per year (e.g., patients 5, 8, 10, 15, 18, and 23 had rates >2% per year). Several hypotheses, including random or selected mutations along with genomic recombination, have been proposed for this observation (*1*,*2*,*9*,*27*). Some patients shed multiple variants simultaneously, suggesting that genomic recombination can occur during replication of SL viruses. Patient 20 was initially excreting iVDPV2 along with SL3; after a few months, only iVDPV3 was detected in his stool. This rapid evolution in the VP1 region to reach iVDPV definition could be explained by crossover of genetic material between the preexisting iVDPV2 and concurrently evolving SL3. The underlying mechanism for iVDPV2 clearance and the predominance of iVDPV3 is unclear in this case, which could be attributable to random chance or host–pathogen interactions.

Patient 17 and her monozygotic twin sister both had SCID. Patient 17 was the only member of the family who shed iVDPV. We suppose that the complete polio immunization history in healthy parents has prevented iVDPV transmission to the sisters. The twin sister had a high risk for iVDPV infection both from OPV administration and transmission from an infected sibling. We cannot rule out a previous iVDPV infection and subsequent spontaneous clearance in the uninfected twin. Also, a risk for future transmission would persist in this situation. Although person-to-person transmission of SL can cause immunity in healthy persons, it might lead to prolonged iVDPV infection in immunodeficient contacts. This risk is high given that PID patients are frequently admitted for IVIG administration or infection control and can transmit the virus to other patients. We advocate regular poliovirus screening of all PID patients, especially in those with CID. More frequent screening could be an appropriate strategy in patients who have direct or indirect contact with known iVDPV excretors. Intensified precautions to prevent transmission by the oral–fecal route should be implemented for poliovirus-excreting (SL or iVDPV) patients during hospital admissions.

**Figure 2:**
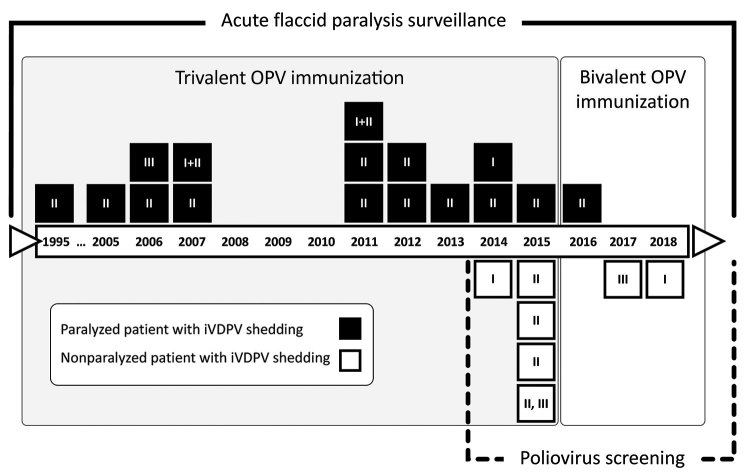
Timeline of Iran’s registry of iVDPV infection, showing the number of patients identified after acute flaccid paralysis or through screening, 1995–2018. The iVDPV detection rate was initially accelerated after implementation of the poliovirus screening program. The switch in vaccination schedule from trivalent to bivalent oral poliovirus vaccine was applied in 2016, leading to a decrease in iVDPV serotype 3 emergence. Two patients excreted iVDPVs with combined serotypes 1 and 2. One patient excreted 2 distinct iVDPVs (serotypes 2 and 3). Numbers in each square indicate the iVDPV serotype. iVDPV, immunodeficiency-associated vaccine-derived poliovirus; OPV, oral poliovirus vaccine.

The fundamental prerequisite for our registry was the implementation of the poliovirus screening program among PID patients. This framework provided the opportunity to diagnose asymptomatic iVDPV excretors and caused a rapid rise in the iVDPV detection rate after 2014 (Figure 2). This acceleration was most dramatic among patients with CID, especially SCID, which increases susceptibility to prolonged and asymptomatic infections (*12*). The rate of asymptomatic shedding was several folds higher in SCID patients than patients with any other PID type; this finding supports the notion that CID patients should receive particular attention in the polio endgame.

Poliovirus screening programs would likely increase the speed of iVDPV detection speed. Although not statistically significant (probably because of the limited study population), this index was lower in patients identified after paralysis than in asymptomatic patients. This trend is consistent with a recent systematic review suggesting that paralysis might be a late manifestation of iVDPV infection (*9*) and highlights the importance of early poliovirus screening for timely diagnosis and treatment in PID patients, mainly in those with adaptive immunity defects (because we did not identify any paralytic patients with defective innate immunity). Although implementing screening might impose higher costs on the health systems, the eradication goal might not be accomplished without a reasonable expenditure on such programs. A case in point is the environmental detection of vaccine-related polioviruses with some features of iVDPV in communities that have not reported any case of wild or vaccine-related poliomyelitis for decades after their switch to IPV (e.g., Finland, Israel, and Switzerland) (*28*–*30*).

For patient 19, PID remained undiagnosed for a long time after its initial manifestations, leading to inappropriate administration of the fourth OPV dose. In addition, patient 23 did not undergo neonatal PID testing and received OPV despite a family history suggestive for primary immunodeficiency. These occurrences might be attributable to insufficient vigilance of primary healthcare providers to signs and risk factors associated with PID. In many countries, neonatal PID screening is in place for early diagnosis of SCIDs and agammaglobulinemia, which assists the physicians in modifying patients’ vaccination scheduling and medical management appropriately (*31*). In addition to implementation of screening programs, improving the awareness of healthcare providers about PID manifestations and the risks associated with OPV administration should be considered as a pivotal part of child healthcare and the polio eradication endgame.

Successful HSCT appears to lead to clearance of iVDPV infection (*22*). Clearing poliovirus infection early after HSCT in patient 15 could be attributed to the recovery phase of innate and cell-mediated immune reconstitution (*22*). Despite appropriate medical therapy, patient 16 never cleared iVDPV2 after >3 years of continuous shedding. HSCT remains the only possible solution for her immunodeficiency and poliovirus infection.

Adjunct to appropriate changes in vaccination strategy, developing new antiviral drugs would be warranted for termination of chronic infections and outbreak control. Pocapavir (V-073) and V-7404 have been suggested as new agents against poliovirus, available for limited, compassionate use for PID patients who are at increased risk for poliovirus infection and are not suitable candidates for HSCT (*15*,*32*). However, the efficacy and safety of these drugs in immunodeficient patients with an established iVDPV infection have not yet been proven.

In conclusion, the emergence of VDPVs remains a serious obstacle in the pathway of polio eradication. Although the recent change in vaccination strategy can prevent the emergence of new cases, unidentified iVDPV excretors still carry the risk for reintroducing viruses from any serotype into the community. Serial screening of PID patients for poliovirus infection is an essential step in the transition to a polio-free world.

Appendix Additional information about vaccine-derived poliovirus infection among patients with primary immunodeficiency and impact of patient screening on disease outcomes, Iran.
