# Supplementary material for: Vaccine-Derived Poliovirus Infection among Patients with Primary Immunodeficiency and Effect of Patient Screening on Disease Outcomes, Iran
Source: Emerg Infect Dis. 2019 Nov;25(11):2005–12. doi: 10.3201/eid2511.190540 (PMC6810208; doi:10.3201/eid2511.190540)
Supplement: Appendix — Additional information about vaccine-derived poliovirus infection among patients with primary immunodeficiency and impact of patient screening on disease outcomes, Iran. [file 19-0540-Techapp-s1.pdf]

Article DOI: https://doi.org/10.3201/eid2511.190540

Vaccine-Derived Poliovirus Infection among Patients with Primary Immunodeficiency and Effect of Patient Screening on Disease Outcomes, Iran

Appendix

Appendix Table. Data summary for all 23 PID patients with iVDPV infection, Iran, 1995–2018*

Patient no.	Report year	Age at iVDPV detection, month/sex	PID	Affected gene	Paralysis	OPV, no. doses	iVDPV serotype	Cleared infection†	Patients outcome	Total replication time, month	Max VP1 divergence, %	Overall evolution rate, %/y
1‡	1995	17/F	HGG	–	Yes	0	2	No	Died	NA	2.2	NA
2	2005	7/M	CID-MHC2	–	Yes	4	2	No	Died	10.5	1.5	1.71
3	2006	10/M	SCID	*RAG2*	Yes	4	2	No	Died	11.0	1.7	1.85
4	2006	15/M	AGG	–	Yes	4	3	Yes	Died	15.1	2.0	1.19
5	2007	5/F	SCID	–	Yes	2	2+1§	No	Died	5.5	2.0<br>1.7	4.36
6	2007	20/M	AGG	–	Yes	4	2	Yes	Survived	31.7	1.2	0.71
7	2011	6/M	CID-NBS	–	Yes	3	2	Yes	Died	21.2	2.1	1.19
8	2011	15/M	AGG	–	Yes	4	2	Yes	Survived	19.2	3.7	2.31
9	2011	25/M	SCID	–	Yes	4	2+1§	No	Died	25.3	3.3<br>1.6	1.56
10	2012	6/M	SCID	–	Yes	1	2	No	Died	8.1	2.3	3.41
11	2012	11/M	AGG	*IGHM*	Yes	3	2	Yes	Survived	11.9	1.5	1.51
12	2013	13/M	–¶	–	Yes	2	2	No	Died	13.4	0.9	0.80
13	2014	10/M	AGG	*IGHM*	Yes	4	1	Yes	Survived	12.1	1.8	1.78
14	2014	9/M	AGG	–	Yes	4	2	Yes	Survived	12.3	1.7	1.66
15	2014	10/M	SCID	–	No	4	1	Yes	Survived	12.4	3.3	3.47
16	2015	10/F	CID-MHC2	*CIITA*	No	4	2	No	Survived	48.9	4.1	0.70
17	2015	6/F	SCID	–	Yes	3	2	No	Died	10.7	1.5	1.68
18	2015	3/M	SCID	*ADA*	No	2	2	No	Died	2.8	0.7	3.00
19	2015	11/F	SCID	*RAG1*	No	4	2	No	Died	11.6	1.1	1.14
20	2015	12/M	AGG	–	No	4	2<br>3#	Yes	Survived	15.4<br>21.4	2.4<br>2.7	1.87<br>1.51
21	2016	14/M	AGG	–	Yes	4	2	Yes	Survived	14.4	0.7	0.58

Patient no.	Report year	Age at iVDPV detection, month/sex	PID	Affected gene	Paralysis	OPV, no. doses	iVDPV serotype	Cleared infection†	Patients outcome	Total replication time, month	Max VP1 divergence, %	Overall evolution rate, %/y
22	2017	8/M	SCID-MHC2	*RFXANK*	No	4 (bOPV)	3	Yes	Survived	13.9	1.3	1.12
23	2018	6/M	SCID	*RAG2*	No	2 (bOPV)	1	No	Died	6.0	1.1	2.2

*Raw data for patients 1–14 have been reported previously (*1*). AGG, agammaglobulinemia; bOPV, bivalent oral-attenuated poliovirus vaccine; CID, combined immunodeficiency; CIITA, class ii major histocompatibility complex transactivator gene; HGG, hypo-gammaglobulinemia; iVDPV, immunodeficiency-associated vaccine-derived poliovirus; IGHM, µ-heavy chain gene; OPV, oral-attenuated poliovirus vaccine; PID, primary immunodeficiency; VP1, viral protein 1; SCID, severe combined immunodeficiency; RAG, recombination activating gene; *ADA,* adenosine deaminase gene; *RFXANK,* regulatory factor x associated ankyrin containing gene; NBS, Nijmegen breakage syndrome; MHC2, major histocompatibility class II deficiency.
†Patients were considered to clear the infection if they had poliovirus negative stool before death or last follow-up.
‡Patient 1 received only inactivated poliovirus vaccine (IPV) and the root of infection was considered transmission from an OPV recipient contact.
§The isolated virus was a mixture of two virus serotypes.
¶PID type was not determined because the patient died before complete workup.
#Two distinct population of the virus was isolated in this patient.

Reference

1. Shaghaghi M, Shahmahmoodi S, Abolhassani H, Soleyman-Jahi S, Parvaneh L, Mahmoudi S, et al. Vaccine-derived polioviruses and children with primary immunodeficiency, Iran, 1995–2014. Emerg Infect Dis. 2016;22:1712–9. PubMed http://dx.doi.org/10.3201/eid2210.151071
